# Asymmetric visual capture of virtual sound sources in the distance dimension

**DOI:** 10.3389/fnins.2022.958577

**Published:** 2022-09-01

**Authors:** Pavel Zahorik

**Affiliations:** ^1^Department of Otolaryngology and Communicative Disorders, Heuser Hearing Institute, University of Louisville, Louisville, KY, United States; ^2^Department of Psychological and Brain Sciences, University of Louisville, Louisville, KY, United States

**Keywords:** visual capture, ventriloquist effect, sound localization, spatial hearing, multisensory, distance, perception, computational model

## Abstract

Visual capture describes the tendency of a sound to be mislocalized to the location of a plausible visual target. This effect, also known as the ventriloquist effect, has been extensively studied in humans, but primarily for mismatches in the angular direction between auditory and visual targets. Here, visual capture was examined in the distance dimension using a single visual target (an un-energized loudspeaker) and invisible virtual sound sources presented over headphones. The sound sources were synthesized from binaural impulse-response measurements at distances ranging from 1 to 5 m (0.25 m steps) in the semi-reverberant room (7.7 × 4.2 × 2.7 m^3^) in which the experiment was conducted. Listeners (*n* = 11) were asked whether or not the auditory target appeared to be at the same distance as the visual target. Within a block of trials, the visual target was placed at a fixed distance of 1.5, 3, or 4.5 m, and the auditory target varied randomly from trial-to-trial over the sample of measurement distances. The resulting psychometric functions were generally consistent with visual capture in distance, but the capture was asymmetric: Sound sources behind the visual target were more strongly captured than sources in front of the visual target. This asymmetry is consistent with previous reports in the literature, and is shown here to be well predicted by a simple model of sensory integration and decision in which perceived auditory space is compressed logarithmically in distance and has lower resolution than perceived visual space.

## Introduction

Auditory-visual interaction has been extensively studied in directional space. In general, the visual system provides superior directional accuracy and resolution, and therefore dominates the perceived direction of sound-producing objects. The dominance is strong enough to produce illusory percepts, such as the well-known ventriloquist situation, where the sound is localized to a plausible visual target even though that target does not actually produce the sound. The ventriloquist’s illusion can influence sound sources separated from visual targets by as much as 55 degrees (Thurlow and Jack, 1973), appears to be strengthened by temporal synchrony between auditory and visual targets (Jack and Thurlow, 1973), but is unaffected by either attention to the visual distracter or feedback provided to the participant (Bertelson et al., 2000). Cortical level mechanisms have been shown to underlie the illusion (Bonath et al., 2007; Callan et al., 2015), which, along with associated aftereffects, suggest a type of short-term plasticity of perceived auditory space mediated by visual input (Recanzone, 1998).

Much less is known regarding auditory-visual interaction in the distance dimension. Pioneering work by Gardner (1968) has suggested an even stronger visual dominance, where sound in anechoic space is always localized in depth to the nearest plausible visual target. Termed the “proximity-image effect,” Gardner (1968) demonstrated complete visual dominance over a range of 9 m between the more distance sound source and the visual target. It is important to note, however, that the auditory distance information available to listeners in these experiments was impoverished due to the use of an anechoic environment. In this type of environment, reverberant sound energy is effectively removed, which in turn removes an important acoustic cue to source distance, namely, the ratio of direct to reverberant sound energy (Zahorik et al., 2005).

Given these facts, Mershon et al. (1980) examined the proximity-image effect under more natural, semi-reverberant acoustical conditions, reasoning that the proximity-image effect may be due largely to auditory distance localization inaccuracies resulting from the anechoic conditions of Gardner’s experiments. To test this reasoning, they visually presented observers with a realistic looking “dummy” loudspeaker in a semi-reverberant room and then played long duration (5-s) noise signals from a loudspeaker occluded from the observer’s view either closer or farther away than the dummy loudspeaker. Ninety percent of the observers (*n* = 441) reported that the sound stimulus appeared to originate from the position of the dummy loudspeaker. Comparing this result to an anechoic condition in which 94% of observers (*n* = 96) reported that the noise stimulus appeared to originate from the dummy loudspeaker, it was concluded that the proximity-image effect operates with nearly the same strength in reverberant conditions as it does in anechoic conditions. It is also interesting to note that although the proximity-image effect was found to be the strongest when the dummy loudspeaker was closer than and the actual sound source, there was also some evidence of the effect in the reversed situation (dummy farther than actual sound source). Thus, there is evidence of a somewhat more general form of visual “capture” of auditory sources in the distance dimension, perhaps related to the angular direction capture reported in studies of ventriloquism effects. More recent results seem to support this hypothesis. Hládek et al. (2021) demonstrate that the strength of visual capture is effectively independent of distance on a logarithmic scale, and that visual capture in distance, like capture in direction, also produces aftereffects.

Computational modeling efforts by Alais and Burr (2004) have fundamentally changed the way the ventriloquist illusion for directional mismatches is viewed conceptually. Previous to their innovative work, the illusion was viewed as a “winner take all” example of visual encoding of spatial information. Their modeling efforts for the ventriloquist illusion instead suggest a probabilistic view, where under most circumstances, the visual encoding of space is simply more reliable. Alais and Burr (2004) confirm this hypothesis by demonstrating that auditory directional encoding can become dominant when directional information from vision is intentionally made less reliable by blurring the visual stimuli. An additional and important prediction from Alais and Burr’s (2004) model is that the precision with which objects are localized in space is always better with multimodal input (auditory + visual) than with unimodal input (visual alone or auditory alone).

Mendonça et al. (2016) applied this type of probabilistic explanation to visual capture in the distance dimension by evaluating a number of different probabilistic models. Although in general, this approach can explain situations both where visual capture in distance is (Gardner, 1968, 1969; Mershon et al., 1980) and is not observed (Zahorik, 2001; Calcagno et al., 2012), the models evaluated by Mendonça et al. (2016) do not incorporate a fundamental aspect of perceived auditory space: that perceived distance is non-linearly related to physical distance. Best evidence suggests that perceived distance is instead logarithmically related to physical sound source distance (see Zahorik et al., 2005 for a meta-analysis of the auditory distance perception literature).

**FIGURE 1:**
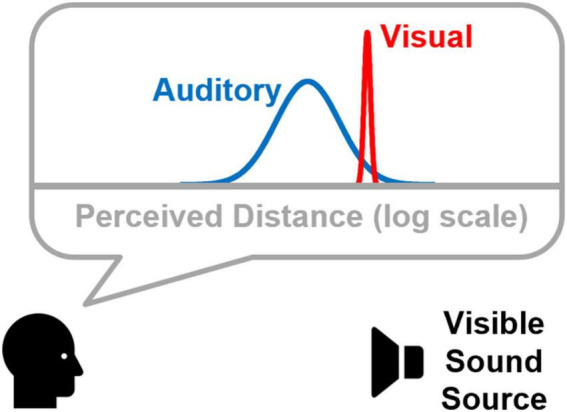
Conceptual framework showing probabilistic distributions of perceived auditory and visual target distances. Note that both are represented on a logarithmic distance axis, and that perceived auditory distance both underestimates the physical distance to the sound source and is less precise. These aspects of the framework were motivated by results previous data sets (Zahorik, 2002; Anderson and Zahorik, 2014).

The purpose of this study is to extend probabilistic modeling of visual capture in distance by including consideration of the logarithmic relationship between perceived distance and physical distance, particularly in the auditory modality. Figure 1 shows a conceptualization of this space, where auditory distance percepts are less precise than visual distance percepts, and they systematically underestimate physical distance. A probabilistic model based on this conceptualization is then used to predict results from a psychophysical experiment in which participants judge whether or not the distance of a virtual sound source matches a visual target. Absolute distance estimates to both visual and (virtual) auditory targets were also collected and used to model the accuracy of auditory and visual distance percepts (e.g., distribution means in Figure 1). Estimates of auditory and visual distance precision (e.g., distribution variances in Figure 1) were taken from data reported by Anderson and Zahorik (2014). Results from this model (M3) are compared to results from two other model variants that represent distance on a linear rather than logarithmic scale. One model variant (M1) specifies auditory and visual distribution variances independently of linear distance. The second variant (M2) scales distribution variances with increasing linear distance.

## Materials and methods

### Subjects

Eleven volunteers (4 male, 7 female; age range 18.1–19.4 years) participated in the psychophysical experiment. All had self-reported normal hearing and normal vision. All procedures involving human subjects were approved by the University of Louisville and University of California–Santa Barbara Institutional Review Boards.

### Testing environment

The experiment was conducted in a large office space (7.7 × 4.2 × 2.7 m^3^) with carpeted floor, painted gypsum board walls, and a drop acoustical tile ceiling. The room had an average background noise level of 31 dBA, and a broadband reverberation time of approximately 0.6 s. The room was illuminated by ceiling-mounted fluorescent lighting (approximately 500 lux) typically used in office spaces. The listener was seated at one end of the room, approximately 1 m in front of the rear wall, and 2.1 m from the side walls. The experiment required two measurement phases in the test environment. The first phase measured the acoustical responses for various sound source distances at the ears of a single listener. These measurements were used to construct a virtual auditory space (VAS) used for subsequent phase two testing. Details of the VAS procedure are described in the next section. The second phase of the experiment measured listener’s judgments of auditory/visual distance and coincidence in distance, using a real visual target (the measurement loudspeaker), and VAS to plausibly reproduce auditory targets independent of visual target location.

### Auditory stimuli and virtual auditory space techniques

A VAS technique, fundamentally similar to that described by Zahorik (2002), was used to present virtual sounds over headphones at distances ranging from 1 to 5 m directly in front of the listener at ear height. To construct the VAS, seventeen binaural room impulse responses (BRIRs) were measured in the testing environment from distances ranging from 1 to 5 m in 0.25 m steps. The sound source was a small, full-range loudspeaker (Micro-spot, Galaxy Audio) placed on a stand at ear level (134 cm above the floor) powered by a high-quality amplifier (D-75, Crown). Miniature electret microphones (Sennheiser KE4-211-2) were placed in the ear canals (blocked-meatus configuration) of a single participant (the author), who did not participate in subsequent psychophysical testing. Previous work concludes that individualized BRIR measurements are not critical for simulating auditory distance (Zahorik, 2000). Maximum-length sequence (MLS) system identification techniques (Rife and Vanderkooy, 1989) were used to measure and derive the BRIRs. The measurement period was 32767 samples at a sampling rate of 44.1 kHz. To improve the signal-to-noise ratio of the measurements, 20 measurement periods were averaged for each distance. Post-averaging, the poorest measurement signal-to-noise ratio was 48 dB (C-weighted), which occurred at 5 m. The MLS measurement technique was implemented in Matlab (Mathworks, Inc.) using a high-quality digital audio interface (CardDeluxe, Digital Audio Labs). In order to equalize for the response of the headphones (Sennheiser HD 410 SL) used in VAS, the impulse responses of the left and right headphones were also measured using similar MLS techniques.

The source signal was a brief sample of broadband Gaussian noise, 100 ms in duration (1 ms rise/fall cosine gate). Independent samples were drawn for each stimulus presentation. No loudspeaker equalization was implemented, so the spectrum of the source signal was shaped by the loudspeaker response characteristics, which limited the bandwidth to between 150 Hz and 18 kHz. All auditory signal processing implemented using MATLAB software (Mathworks Inc., Natick, MA, United States).

### Visual stimuli

The visual stimulus was the (single) measurement loudspeaker, viewed binocularly, and placed at distances ranging from 1 to 4.5 m directly in front of the observer.

### Procedure

The experiment consisted of three psychophysical measurement phases: Yes/No judgments of Auditory/Visual target coincidence, absolute judgments of virtual auditory target distance, and absolute judgments of visual target distance. All participants completed all three phases of the experiment, in the order listed. Participants were not provided with any response feedback in any phase of the experiment.

#### Coincidence judgments

Participants were presented with a visual target and a virtual auditory target from either the same or different distances, and were instructed to respond whether the perception was of matching (coincident) auditory and visual target distance, or not. Participants were told that the sound could actually originate from the loudspeaker visual target, or be a virtual sound that was produced by the headphones, even though in actuality all sounds were virtual. This was done to avoid potential biases that could result if participants knew that physical coincidence was impossible. This was also the rationale for testing coincidence prior to absolute judgments, where exposure to virtual sounds alone was known to participants. To facilitate good registration between auditory and visual targets, head orientation was monitored using a laser pointer. The pointer was mounted to the headband of the headphones and pointing straight ahead. Participants were instructed to keep the laser pointer aimed at a 2-cm circular target affixed to the front of the loudspeaker visual target. Compliance with this instruction ensured orientation remained fixed within one degree. Head orientation compliance was monitored by an experimenter, who controlled the initiation of each trial and entered the participant’s coincidence responses on a keypad. Head position was monitored because the VAS was not updated in response to head movements. Thus, significant head movements could have caused the auditory stimulus to not be perceived in the direction as the visual stimulus. Testing was conducted blocked by visual target distance. For each visual target distance, 11 virtual auditory targets distances surrounding the visual target were tested 30 times, in randomized order. Visual target block order was also randomized. Participants completed this portion of the experiment in approximately 30 min.

Coincidence judgments were analyzed both on an individual-subject basis and pooled across all subjects. Both types of analyses first computed the proportion of coincidence responses, *pc*, observed for each auditory target distance, and then recorded the maximum value of this quantity, *pc*_*max*_, that occurred across all auditory target distances. For individual subject analyses, *pc* and *pc*_*max*_ were computed on an individual-subject basis. For pooled analyses, *pc* and *pc*_*max*_ were computed on data pooled across all subjects. Both types of analyses then combined *pc* and *pc*_*max*_ into a single index of discriminability metric, *d′*, as follows:

$$d^{\prime }=z\,\left(p\,c_{m\,a\,x}\right)-z\,\left(p\,c\right)$$

where *z*(*pc*) and *z*(*pc*_*max*_) represent *pc* and *pc*_*max*_ converted to standard normal deviates (*z*-scores). Conceptually, the *d′* metric represents the degree of perceptual mismatch (discriminability) between auditory and visual targets referenced to the point that produced the most coincidence responses (*pc*_*max*_).

#### Auditory distance judgments

Participants made absolute judgments of virtual auditory target distances in the absence of any plausible visual targets. Virtual auditory target distances ranged from 1 to 5 m in 0.5 m steps. Head movement was monitored using procedures identical to those used for coincidence judgments, except that the circular target to which the participant was instructed to aim the pointer was mounted on the back wall of the testing room. Participants could use distance units with which they had the most familiarity (e.g., either feet/inches, or meters/centimeters), and were instructed to be as precise as possible with their estimates. A single distance judgment from each distance was recorded. Presentation order of distances was randomized. Participants completed this portion of the experiment in approximately 5 min.

#### Visual distance judgments

Judgments of absolute distance were collected for visual target distances of 1, 2, and 3 m in the absence of auditory targets. Procedures were identical to those used for auditory distance judgments, except that the head orientation was fixed to the loudspeaker visual target. Participants completed this portion of the experiment in approximately 2 min.

### Coincidence models

**FIGURE 2:**
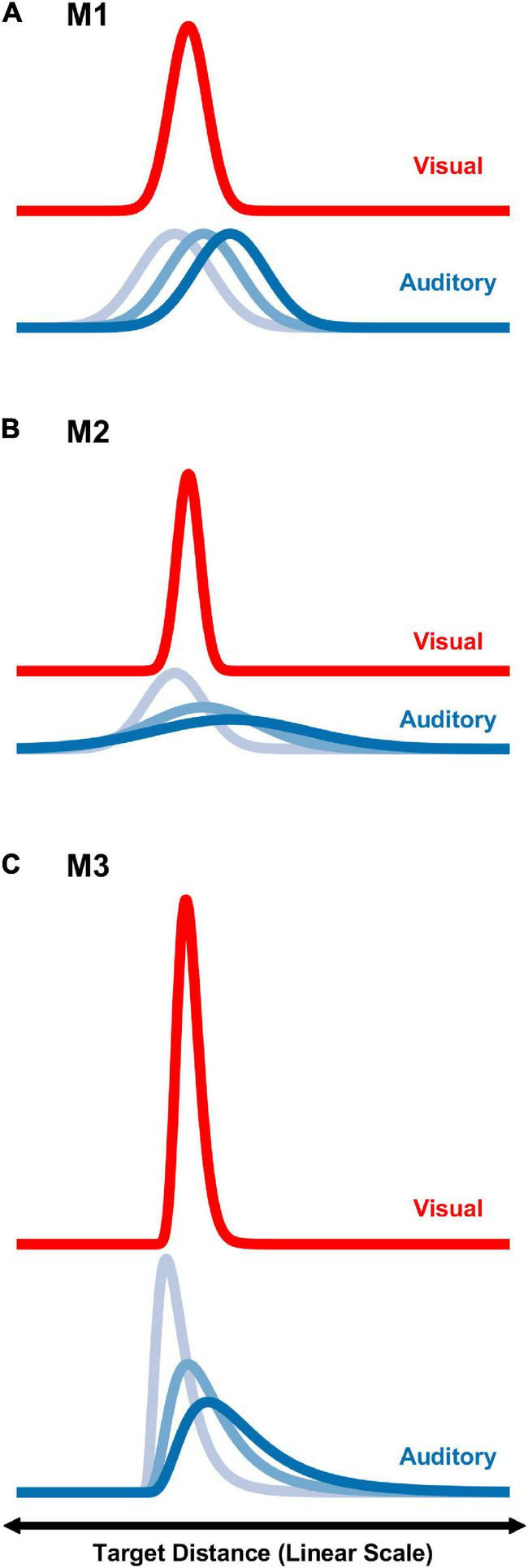
Graphical depictions of the three modeling strategies, M1–M3 **(A–C)**, on a linear distance scale. For each strategy, distributions associated with a visual target at 1.5 m, and auditory targets at 1.5, 3, and 4.5 m are shown. See text for additional details.

To predict the results of the psychophysical distance coincidence judgments three different computational models were evaluated. Model 1 (M1) considered perceived distance on a linear scale with constant auditory and visual distance variances. Model 2 (M2) also considered perceived distance on a linear scale, but with auditory and visual variances that scaled with mean distance. Model 3 (M3) considered perceived distance on a logarithmic scale. All models conceptualize auditory/visual coincidence judgments as the result of a comparison of internal distributions of perceived auditory distance and perceived visual distance. The degree of overlap in the two distributions was then be used in a probabilistic sense to estimate judgments of coincidence. The shapes of the distributions are normal in either linear (M1 and M2) or logarithmic distance space (M3). Figure 2 displays conceptual examples of M1, M2, and M3.

From signal detection theory, the distribution overlap can be evaluated using the sensitivity index, *d′*. Here, we define *d′* as

$$d^{\prime }=\frac{\left|\mu_{a\,u\,d}-\mu_{v\,i\,s}\right|}{\sqrt{\sigma_{a\,u\,d}^{2}+\sigma_{v\,i\,s}^{2}-2\,r\,\sigma_{a\,u\,d}\,\sigma_{v\,i\,s}}}$$

where *μ_*aud*_* and *μ_*vis*_* are the means of the perceived auditory and visual distributions, σ^2^*_*aud*_* and σ^2^*_*vis*_* are the variances. The parameter *r* represents the correlation between perceived auditory and visual distance. Although *d′* is often computed assuming independence of the two distributions, and hence *r* = 0, we felt that it was appropriate in this case to consider potential non-zero correlation between perceived auditory and visual distances, given the coincidence judgment paradigm with concurrently presented auditory and visual stimuli.

For all models, data from auditory and visual absolute distance judgments in this study were used to estimate *μ_*aud*_* and *μ_*vis*_*. These estimates were based on power functions fit to the absolute distance judgment data, consistent with methods used in previous studies (Zahorik, 2002; Anderson and Zahorik, 2014). The power functions had the form *y* = *kx^a^*, where *a* and *k* are estimated exponent and constant values, *x* is physical distance (linear scale) and *y* is judged distance. Therefore, for auditory we assume

$$\mu_{a\,u\,d}=k\,x_{a\,u\,d}^{a}$$

and for visual we assume

$$\mu_{v\,i\,s}=k\,x_{v\,i\,s}^{a}$$

Based on physical auditory and visual target distances, *x*_*aud*_ and *x*_*vis*_, and power function fit parameters *a* and *k*. Although both *a* and *k* were estimated independently from the auditory and visual absolute distance judgment data in this study, for the coincidence models, we allowed the *k*_*aud*_ to be a free parameter, using the rationale that visual distance percepts may have served as an absolute reference for the auditory distance judgments under multimodal stimulus conditions.

Specifics of *d′* calculations for each model, including variance parameter estimate strategies, and translation to a logarithmic distance scale, are described in the following subsections. Models were fit to the coincidence estimate data using a non-linear least squares procedure, (lsqcurvefit) in Matlab (Mathworks, Inc.), with the constraint 0 ≤ *r* ≤ 1.

#### M1: Linear distance scale, constant variance

For this model, the variances, *σ^2^_*aud*_* and *σ^2^_*vis*_* were considered as fixed constant values. These values were based on absolute distance judgment data from previously published work (Anderson and Zahorik, 2014) using a large samples of participants (auditory *n* = 62, visual *n* = 45). In that work, *σ_*aud*_* and *σ_*vis*_* were found to be multiplicative factors of distance in meters; 10^0.23^ = 1.6982 and 10^0.15^ = 1.4125, on average for auditory and visual distance, respectively. For M1, however, we chose to estimate *σ_*aud*_* and *σ_*vis*_* at the perceived distance associated with the mean target distance for the current study, 3 m. Therefore, at the fixed value of *x* = 3 and we have

$$\sigma_{a\,u\,d}=1.6982\,k\,x_{a\,u\,d}^{a}=1.6982\,k\,3_{a\,u\,d}^{a}$$

and

$$\sigma_{v\,i\,s}=1.4125\,k\,x_{v\,i\,s}^{a}=1.4125\,k\,3_{v\,i\,s}^{a}$$

where *a* and *k* were estimated independently from the auditory and visual absolute distance judgment data from this study. The correlation, *r*, between perceived auditory and perceived visual distance was also a free parameter in this coincidence model.

#### M2: Linear distance scale, scaled variance

This coincidence model was identical to M1, but allowed for the variance estimates of auditory and visual distance percepts to scale with distance. This allowance more accurately reflects the observation from previous work (Anderson and Zahorik, 2014) that the judgment variability using an absolute distance judgment paradigm increases with increasing distance. Thus, for M2, we modified *σ_*aud*_* and *σ_*vis*_* as defined in M1, by allowing them to scale with perceived distance over the range of auditory and visual target distances tested. Specifically,

$$\sigma_{a\,u\,d}=1.6982\,k\,x_{a\,u\,d}^{a}$$

and

$$\sigma_{v\,i\,s}=1.4125\,k\,x_{v\,i\,s}^{a}$$

where *σ_*aud*_* varied as a function of auditory target distance, 0 < *x*_*aud*_ < 10 m, and *σ_*vis*_* was considered for each of the visual target distances evaluated, *x*_*vis*_ = 1.5, 3.0, or 4.5 m. Like M1, *k*_*aud*_ and *r* were free parameters in M2.

#### M3: Logarithmic distance scale (scaled variance)

M3 was identical to M2, except that it considered distance on a logarithmic scale instead of a linear scale. Thus, the assumptions of normal distributions of perceived auditory and visual distance were qualified to apply to log-distance, rather than linear distance. This assumption is consistent with the observations reported in Anderson and Zahorik (2014); that absolute distance judgments of auditory and visual targets appear to be normally distributed in logarithmic space. To facilitate comparison with results from M1 and M2, it was noted that normal distributions in logarithmic space are equivalent to log-normal distributions in linear space, and therefore M3 was represented in linear space assuming log-normal distributions of perceived auditory and visual distance. Log-normal distributions, by definition, have the property that the variance scales with the mean. The transformation process to log-normal distributions was accomplished by first representing the mean (*m*) and standard deviation (*s*) parameters for *d′* in (natural) logarithmic space,

$$m_{a\,u\,d}=l\,n\,\left(k\,x_{a\,u\,d}^{a}\right)$$

$$m_{v\,i\,s}=l\,n\,\left(k\,x_{v\,i\,s}^{a}\right)$$

$$s_{a\,u\,d}=l\,n\,\left(1.6982\right)$$

$$s_{v\,i\,s}=l\,n\,\left(1.4125\right)$$

and then computing the parameters log-normal distributions to represent auditory and visual perceptual spaces

$$\mu_{a\,u\,d}=\text{exp}\,\left(m_{a\,u\,d}+\frac{s_{a\,u\,d}}{2}\right)$$

$$\mu_{v\,i\,s}=\text{exp}\,\left(m_{v\,i\,s}+\frac{s_{v\,i\,s}}{2}\right)$$

$$\sigma_{a\,u\,d}=\sqrt{\left[\exp\left(s_{a\,u\,d}^{2}\right)-1\right]\,e\,x\,p\,\left(2\,m_{a\,u\,d}+\sigma_{a\,u\,d}^{2}\right)}$$

$$\sigma_{v\,i\,s}=\sqrt{\left[\exp\left(s_{v\,i\,s}^{2}\right)-1\right]\,e\,x\,p\,\left(2\,m_{v\,i\,s}+\sigma_{v\,i\,s}^{2}\right)}$$

As in both M1 and M2, *a*_*aud*_, *a*_*vis*_, and *k*_*vis*_ were estimated from the absolute distance judgment data obtained in this study, and standard deviation parameters, *s*_*aud*_ and *s*_*vis*_, were based on data from Anderson and Zahorik (2014), and *k*_*aud*_ was a free parameter. Log-normal distribution parameters were then entered into the *d′* equation, where *r* was once again a free parameter.

## Results

### Acoustical cues to distance

**FIGURE 3:**
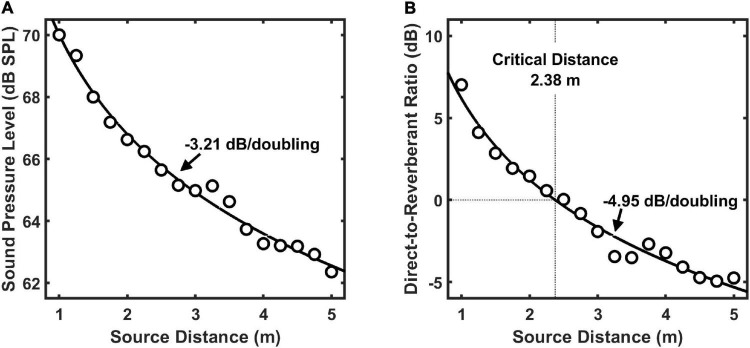
Broadband sound pressure level **(A)** and direct-to-reverberant energy ratio **(B)** for the test stimulus as a function of sound source distance in the test environment. For each acoustic cue, functions were fit to the data to describe the decibel (dB) loss of that cue per doubling of source distance. The critical distance for the test environment, which is the distance at which direct and reverberant energies are equal, was 2.38 m.

Sound pressure level and direct-to-reverberant energy ratio measures as a function of distance are shown in Figure 3. These measures were derived from the BRIR measurements conducted for VAS simulation, and are known to be primary acoustic cues to distance (Zahorik et al., 2005; Kolarik et al., 2016). Their orderly change with distance indicates that the acoustical environment used for testing provided good acoustic information for auditory distance estimation.

### Absolute auditory and visual distance judgments

**FIGURE 4:**
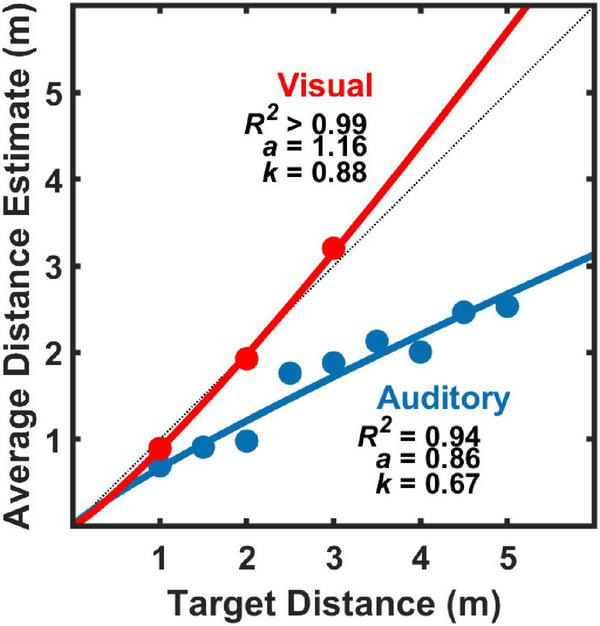
Average (geometric mean) estimates of target distance for auditory stimuli alone and for visual stimuli alone (*n* = 11) as a function of physical target distance For each modality, the data were fit with power functions of the form: *y* = *kx^a^*, where *y* is the average distance estimate, *x* is the physical target distance. Parameter estimates for *k* and *a* are shown for each modality, as is the proportion of distance estimate variance explained by the power function fit (*R*^2^).

Figure 4 displays group average results (*n* = 11) for absolute distance judgments to auditory targets alone (blue symbols) or visual targets alone (red symbols). Power function fit parameters *a* and *k* are shown for each fit, as well as *R*^2^ to assess goodness of fit. Consistent with past results, power functions were found to be good fits to the data (Zahorik, 2002; Zahorik et al., 2005; Anderson and Zahorik, 2014), visual distance perception was found to be highly accurate (Da Silva, 1985; Anderson and Zahorik, 2014), and auditory distance perception was found to be less accurate, with systematic underestimation as target distance increased (Zahorik, 2002; Anderson and Zahorik, 2014).

### Coincidence judgments and modeling results

**FIGURE 5:**
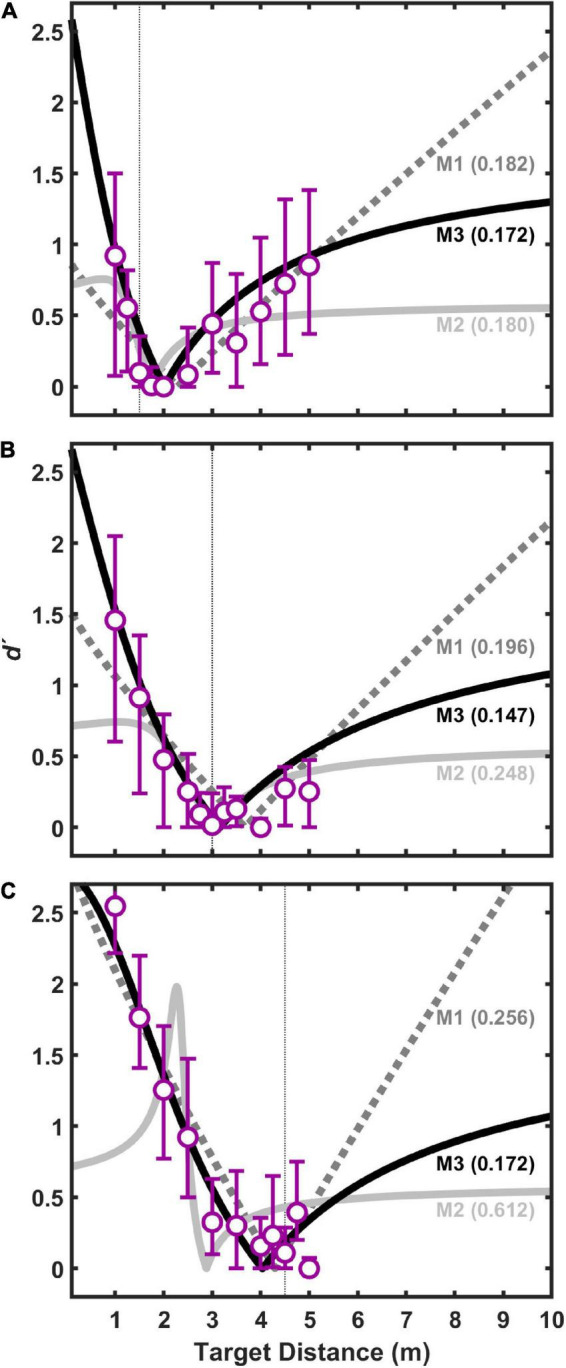
Index of discriminability, *d′*, for pooled data from all subjects as a function of auditory target distance for three difference visual target distances: 1.5 m **(A)**, 3.0 m **(B)**, and 4.5 m **(C)**. Each point is based on 330 trials (30 responses/stimulus × 11 subjects). Bars indicate 95% confidence intervals (*n* = 11) estimated using a bootstrapping procedure. For each visual target distance, the data were fit with three different models, M1–M3, which are shown conceptually in Figure 2. Each fit had two free parameters: the constant, *k*_*aud*_, in the auditory distance power function, and *r*, which is the correlation between internal estimates of auditory and visual distance (see text for details). Goodness of fit for each model was assessed using a root mean squared (RMS) error metric (shown in parentheses for each model). Smaller RMS errors indicate better fits.

Figure 5 displays *d′* results for pooled (*n* = 11) coincidence judgments to visual target distances of 1.5 m (Figure 5A), 3 m (Figure 5B), and 4.5 m (Figure 5C) as a function of auditory target distance. Values of *d′* near zero indicate perceived coincidence of auditory and visual targets, and therefore strong visual capture if auditory and visual target are displaced in physical distance. This result also suggests that our procedures, including those implemented to limit head movements during the experiment, were sufficient to produce good registration between auditory and visual targets. In general, the pattern of results is consistent with the “proximity image effect” (Gardner, 1968; Mershon et al., 1980), where sound sources closer than a plausible visual target are more likely to be judged as non-coincident (larger *d′* value) than sound sources farther than the visual target. Here, the effect is visible as an asymmetric pattern of *d′* values as a function of (linear) distance. The effect is most evident for visual target distances of 1.5 and 3.0 m (Figures 5A,B). The effect is less evident in the data at the 4.5 m visual target distance (Figure 5C) because the test environment did not permit many auditory target distances farther than visual target in this case. Concentrating on the data for visual target distances of 1.5 and 3.0 m (Figures 5A,B), it is also noteworthy that the points of strongest visual capture (lowest *d′* values) were generally for auditory targets at distances slightly greater than visual target distances. This is consistent with the underestimation of auditory distance shown here in Figure 4, and with results from a variety of previous studies of auditory distance perception (see review and analysis in Zahorik et al., 2005).

Also displayed in Figure 5 are the results of the three models (M1, M2, and M3) fit to the coincidence data shown. For each model, *a*_*aud*_, *a*_*vis*_, and *k*_*vis*_ were estimated from the group-average absolute distance judgment data obtained shown in Figure 4, and *k*_*aud*_ and *r* were estimated parameters based on the coincidence data pooled across listeners. Root-mean-squared (RMS) error in *d′* units is displayed in Figure 5 for each model fit. In all cases, M3 produced the lowest RMS errors, and was therefore the best fit to the data. M1 and M2 were generally poorer fits to the data, often with marked prediction inaccuracies for distances much closer and much farther than the visual target distance.

**FIGURE 6:**
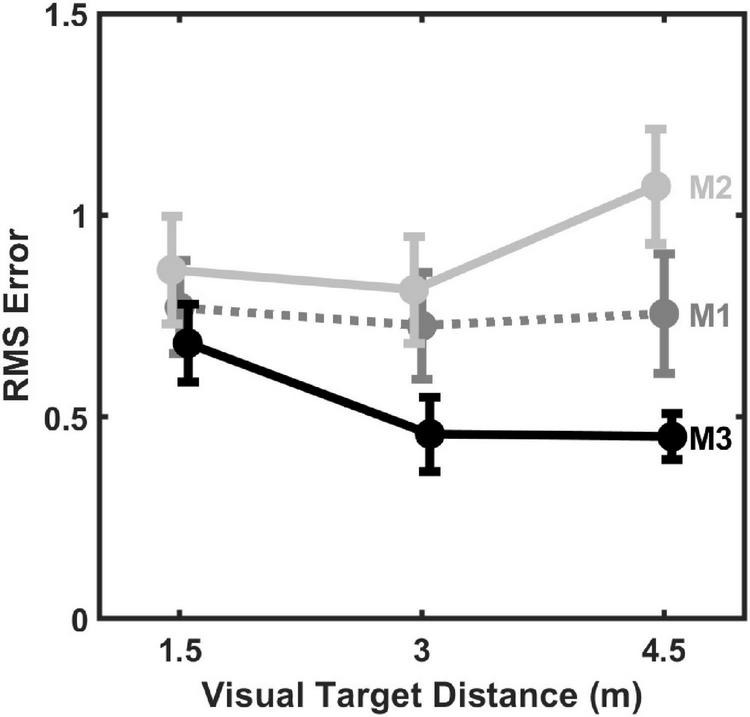
Mean (±1 standard error) RMS error of 2-parameter (*k*_*aud*_, *r*) model fits to individual subject data (*n* = 11) as a function of visual target distance.

The same three models were also fit to coincidence data from individual subjects, using *a*_*aud*_, *a*_*vis*_, and *k*_*vis*_ estimated from individual subject absolute distance judgment data. As before, *k*_*aud*_ and *r* were estimated parameters, but now estimated separately for each individual subject model. Model fit results are summarized in Figure 6, which displays average (mean) RMS error across all subjects for each model fit to individual subject data as a function of visual target distance. From Figure 6 it can be observed that M3 generally produced the lowest fit error, followed by M1, and then M2. Note that these RMS values are higher than those shown in Figure 5 because they are based on fits to individual subject data (30 responses/distance) rather than to data pooled over all subjects (330 responses/distance). Results of a Linear Mixed-Effects Model (LMM) with model type (M1, M2, or M3) and visual target distance (1.5, 3, or 4.5 m) as fixed factors and subject intercept as a random factor confirmed these observations. There was a significant main effect of model type, *F*(2,80) = 13.726, *p* < 0.001, but neither visual target distance, *F*(2,80) = 1.240, *p* = 0.295, nor the distance × model interaction were significant, *F*(4,80) = 1.603, *p* = 0.182. *Post hoc* testing of the significant model type main effect revealed that M3 error was lower than M1 error, *t*(80) = 2.987, *p* = 0.004, which in turn was lower than M2 error, *t*(80) = 2.243, *p* = 0.014 (*p*-values adjusted for multiple comparisons using the Holm–Bonferroni method). Thus overall, M3 objectively produced the best fits to individual data.

**FIGURE 7:**
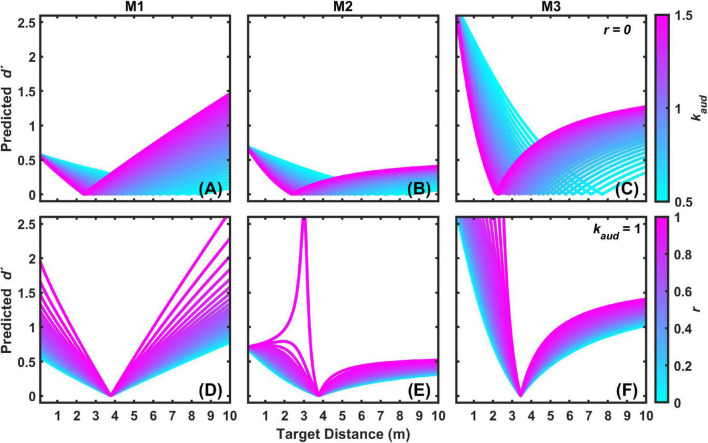
Examples of predicted *d′* functions for the 3 m visual target distance for each 2-parameter model (M1–M3) as a function of *k*_*aud*_ with *r* = 0 **(A–C)**, and as a function of *r* with *k*_*aud*_ = 1 **(D–F)**.

M3 also arguably provides the best qualitative fit to the data, because it is the model that most effectively accounts for the asymmetry in coincidence judgments depending on whether the auditory target is closer or farther than the visual target, as can be observed in Figure 5. To better demonstrate this assertion, output from all three models were compared in an exploration of the fitted parameter space. Figure 7 displays an example of this analysis for the 3 m visual target distance. Figures 7A–C show the results of models M1–M3 for varying *k*_*aud*_ with *r* fixed at a value of zero. Figures 7D,E show the results of models M1–M3 for varying *r* with *k*_*aud*_ fixed at a value of 1. In general, *k*_*aud*_ controls the position of function minima on the target distance axis, and *r* controls the “sharpness” of the functions. From Figure 7 it is clear that M3 produces the most pronounced coincidence asymmetry, as well as an orderly progression of function sharpness with increasing *r*, particularly for close distances. Qualitatively similar results were observed for other exploration analyses at visual target distances of 1.5 and 4.5 m (not shown).

### Model variations and further analyses

**FIGURE 8:**
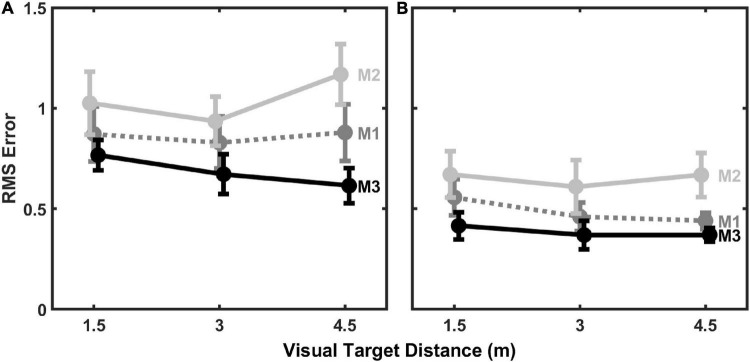
Mean (±1 standard error) RMS error of **(A)** 1-parameter (*k*_*aud*_) model fits and **(B)** 3-parameter (*k*_*aud*_, *r, σ*_*aud*_) model fits to individual subject data (*n* = 11) as a function of visual target distance.

To evaluate the sensitivity of modeling results to the number of estimated parameters in the models, we also evaluated model fits to individual-subject data with 1 and 3 estimated parameters. The 1-paramter models estimated *k*_*aud*_. The 3-parameter models estimated *k*_*aud*_, *r*, and *σ_*aud*_*. Thus, these additional models represent both simplifications (1-parameter) and expansions (3-parameters) from the 2-parameter model results shown in Figures 5–7. Results from these additional model fits are summarized in Figure 8, which displays average (mean) RMS error across all subjects for each model fit to individual subject data as a function of visual target distance. Not surprisingly, the 1-parameter model errors (Figure 8A) are somewhat higher than the 2-parameter errors (Figure 6), which in turn are somewhat higher than the 3-parameter errors (Figure 8B). M3 generally produced the lowest fit error in all cases, however. Results from LMM analyses, analogous to those performed on the 2-parameter model errors, confirm the superiority of M3 for both 1- and 3-parameter models. Significant main effects for model type were observed for both the 1-parameter models, *F*(2,80) = 10.094, *p* < 0.001, and the 3-parameter models, *F*(2,80) = 10.804, *p* < 0.001. The effects of distance and distance × model type interaction were not statistically significant for either 1-parameter or 3-parameter models. *Post hoc* testing (*p*-values adjusted for multiple comparisons using the Holm–Bonferroni method) revealed that for the 1-parameter models, M3 produced the lowest overall error: M3 error was significantly less than M1 error, *t*(80) = 2.200, *p* = 0.016, which is turn was significantly less than M2 error, *t*(80) = 2.288, *p* = 0.025. Similar *post hoc* results were observed for the 3-parameter models, where M3 also produced the lowest overall error: M3 error was significantly less than M1 error, *t*(80) = 1.772, *p* = 0.042, which is turn was significantly less than M2 error, *t*(80) = 2.877, *p* = 0.006.

**FIGURE 9:**
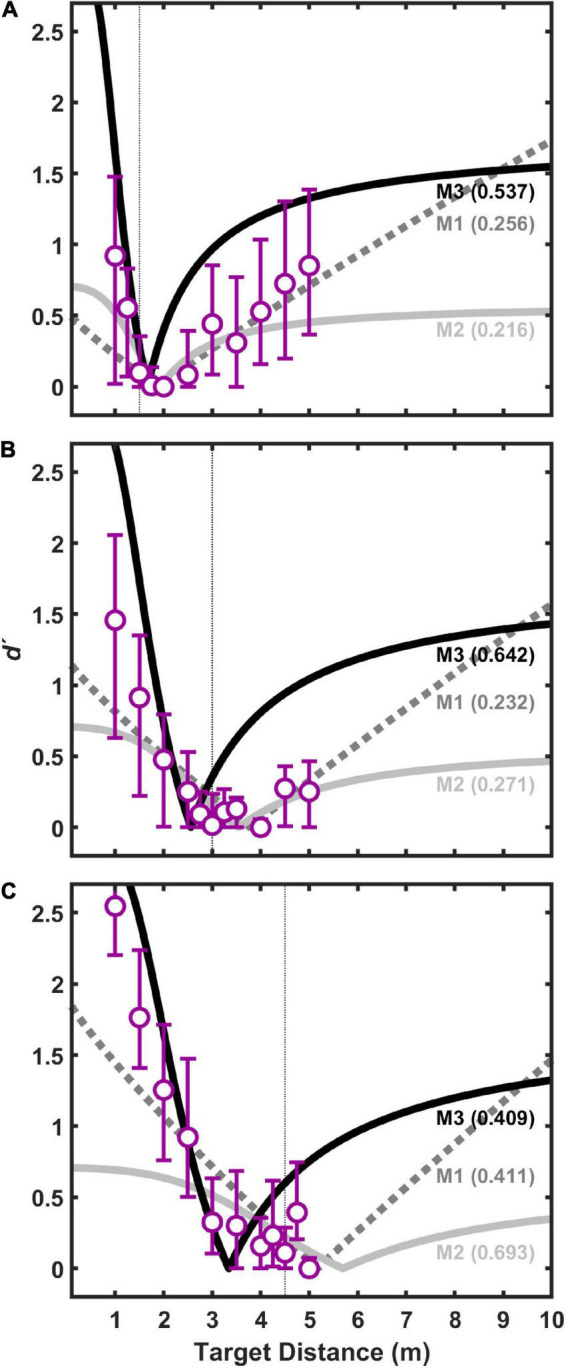
Same (pooled) data for visual target distances of 1.5 m **(A)**, 3.0 m **(B)**, and 4.5 m **(C)** as shown in Figure 5, but fits displayed are for 1-parameter (*k*_*aud*_) models.

**FIGURE 10:**
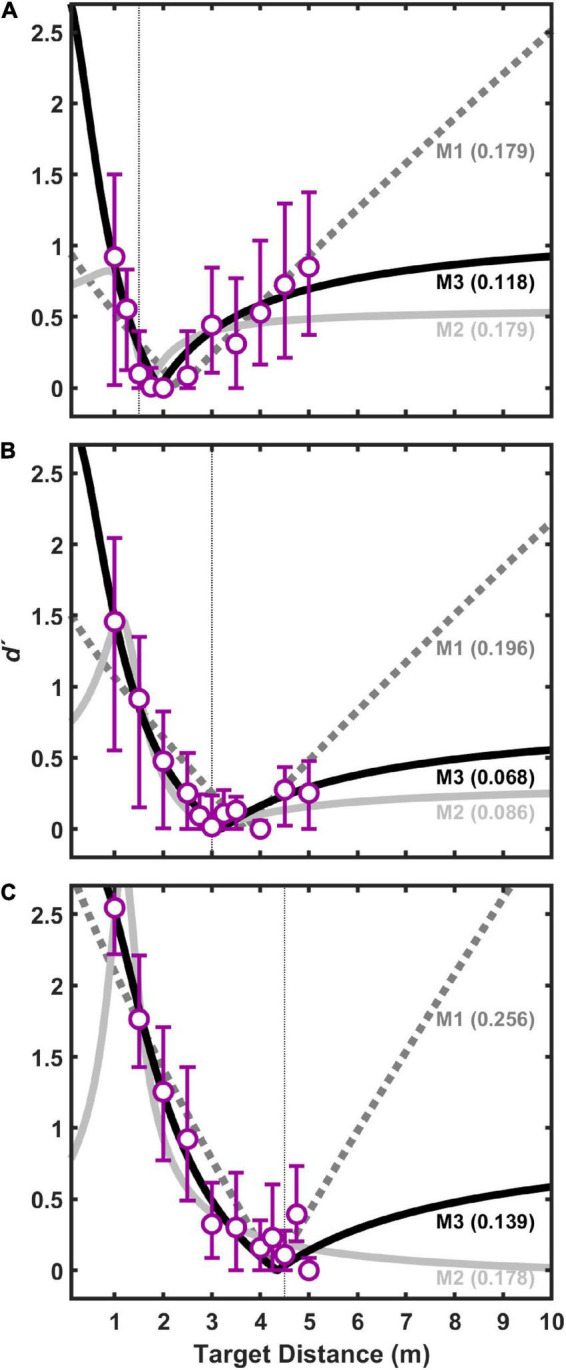
Same (pooled) data for visual target distances of 1.5 m **(A)**, 3.0 m **(B)**, and 4.5 m **(C)** as shown in Figure 5, but fits displayed are for 3-parameter (*k*_*aud*_, *r, σ*_*aud*_) models.

Figures 9, 10 show fit results to the pooled data (same as Figure 5) for the 1-parameter and 3-parameter models, respectively. A clear and large jump in model accuracy can be observed comparing 1-parameter (Figure 9) to 2-parameter (Figure 5) fits. Although more modest gains in accuracy are observed moving from 2-parameter (Figure 5) to 3-parameter (Figure 10) fits, both show a clear superiority of M3.

## Discussion

Results from this study demonstrate robust visual capture of virtual sound sources varying in distance, but the capture was asymmetric: Sound sources behind the visual target were more strongly captured than sources in front of the visual target. This asymmetry is consistent with “the proximity image effect” (Gardner, 1968; Mershon et al., 1980), and is shown to be well predicted by a simple model (M3) of sensory integration and decision in which perceived auditory space is both logarithmically compressed in distance and has lower resolution than perceived visual space. Comparable models that assume a linear distance space (M1 and M2) were less successful at predicting the observed responses of auditory/visual coincidence.

These results are important because they link the seemingly disparate aspects of distance perception and visual capture to potentially common underlying principles that include:

1. Imprecision of auditory distance perception relative to visual distance perception. Elegant modeling work by Mendonça et al. (2016) clearly demonstrates that visual capture in distance is explainable based on the relative imprecision of auditory distance perception relative to visual distance perception. This concept is a fundamental aspect of other probabilistic models of multisensory integration which result in more weight being given to perceptual modalities that produce more precise estimates of a given perceptual quantity (Ernst and Banks, 2002; Alais and Burr, 2004). This principle is also consistent with an increased variability in judgments of absolute distance to auditory targets relative to visual targets, as observed in previous studies (Zahorik, 2001; Anderson and Zahorik, 2014). All models in the current study included this principle.
2. Accurate visual distance perception, but biased auditory distance perception. Although the auditory distance estimates reported in Mendonça et al. (2016) do not show strong bias, most studies of auditory distance perception do, including the current study. Typically, far sources (>∼1 m) are underestimated in distance and close sources (<∼1 m) are overestimated (Zahorik et al., 2005), although there can be substantial variation in the amount of bias. For example, auditory distance judgments are known to be extremely biased in anechoic space, which was the type of space where the proximity image effect was first identified (Gardner, 1968). Conversely, judgments of visual target distances are known to be highly accurate under everyday viewing situations (Da Silva, 1985; Anderson and Zahorik, 2014). All models in the current study therefore included this principle.
3. Logarithmic distance scaling. Conceptualization of distance perception on a logarithmic scale appears to be critical for explaining the asymmetric aspects of the visual capture (i.e., the proximity image effect). The superiority of logarithmic scaling in the current modeling efforts to explain coincidence judgments is particularly important because it provides converging evidence that the underlying perceptual space for distance—particularly auditory distance—is logarithmic. Prior conclusion regarding the logarithmic nature of perceived auditory space come from very different origins: analysis of errors in absolute distance judgments (Anderson and Zahorik, 2014). Thus, the fact that two different phenomena can be predicted by the same idea, that of an underlying logarithmic space of perceived auditory distance, lends strength to the idea. A further rationale for a logarithmic scale of distance is that many of the primary acoustic cues to distance are also logarithmically related to physical distance, such as sound intensity and the ratio of direct to reverberant sound energy (see Zahorik et al., 2005 and Kolarik et al., 2016 for reviews). Modeling efforts by Mendonça et al. (2016) did not consider distance on a logarithmic scale, but their data also did not appear to show strong capture asymmetries in distance. Visual capture work by Hládek et al. (2021) did represent auditory distance on a logarithmic scale, but their work did not include modeling. The M3 model in the current study implemented this logarithmic scaling principle.

An important implication of the logarithmic distance scaling principle is that adequately sampling of a logarithmic distance space may require very large physical spaces. In the current study, for example, this was likely a problem for the condition where the visual target was placed at 4.5 m. In this case, there was very little room beyond the visual target even in linear space for additional auditory sources. That space was trivially small in on a logarithmic scale. As a result, expanded testing of the principles proposed in this study should be conducted in an environment capable of supporting a greater distance range.

The experimental challenges of successfully working with a logarithmic distance space may also explain why Hládek et al. (2021) did not observe asymmetry in their visual capture data consistent with the proximity image effect. Their methodology only displaced auditory and visual targets by ± 30% in distance, and they only measured up to a maximum distance of 203 cm. From the data reported in the current study at a visual target distance of 1.5 m, auditory targets at ±30% of this value also did not produce hugely different estimates of *d′*, roughly 0 and 0.5 for auditory source distances of 1.95 m (+30%) and 1.05 m (−30%). Thus, it would be difficult to make strong conclusions regarding capture asymmetry in the current data as well if only two distances were considered that are relatively close in logarithmic space. This is another demonstration of the need for testing a large range of distances.

In the visual depth perception literature, there has been some debate as to the metric (e.g., linear versus logarithmic) of the underlying perceptual space (Loomis et al., 1992; Wagner, 2012). Given that visual distance perception is typically highly accurate, as seen here in Figure 4, the observed differences between logarithmic and linear spacing can be very small. This is not the case for auditory distance perception, where estimates of auditory distance exhibit strong non-linear compression. Such compression is observed in the current study (Figure 4), and is consistent with many other studies of auditory distance perception (see Zahorik et al., 2005 and Kolarik et al., 2016 for reviews), including some of the few studies that have examined the neural bases of auditory distance perception (Kopčo et al., 2012; Kim et al., 2015).

Finally, the asymmetric nature of visual capture in distance also has implications for the number and choice of estimated parameters in the computational model. From the results of the current study, it is clear that when only the *k*_*aud*_ parameter is allowed to vary, such as shown in Figures 7A–C, the degree of modeled function asymmetry (i.e., “sharpness”) is not a tunable parameter. This likely explains why model fit errors were much higher for the 1-parameter model (Figure 8A) than for the 2-parameter model (Figure 6) that allowed asymmetry tuning. Although the 3-parameter model did provide some additional reduction in fit error (Figure 8B), parsimony suggests that the 2-parameter model may be preferable in this case, because it still provides enough flexibility to model essential aspects of the data. Further study with measurements at more distances will be needed to fully explore the strengths and weaknesses of models that include 3 or more parameters.

## Conclusion

Results from three sets of human psychophysical measurements demonstrate that: (a) absolute judgments of visual distance are generally accurate under everyday viewing conditions, (b) judgments of auditory distance are logarithmically compressed under the same everyday conditions, and (c) judgments of auditory/visual coincidence (i.e., visual capture) are asymmetric, such auditory sources farther than a visual target are more likely to be judged as coincident than are auditory sources closer than the visual target. Coincidence judgment data were well predicted by a computational model where perceived auditory distance is both less precise and less accurate than perceived distance, and distance is represented on a logarithmic scale. Taken together, these results are significant from a basic science perspective because they represent converging evidence that, particularly in the auditory modality, the scale of perceived distance is logarithmic. These results may also be of practical significance for applications such as auditory/visual rendering because they suggest that there are many situations where auditory distance may be rendered with less precision and yet have minimal impact on spatial perception, such as at greater distances and when sound-producing objects are visible.

## Data availability statement

The original contributions presented in this study are publicly available and can be found here: https://doi.org/10.5281/zenodo.6999182. Further inquiries can be directed to the corresponding author.

## Ethics statement

The studies involving human participants were reviewed and approved by University of California–Santa Barbara IRB and University of Louisville IRB. The patients/participants provided their written informed consent to participate in this study.

## Author contributions

PZ designed, conducted, and analyzed the data, wrote the manuscript, contributed to the article, and approved the submitted version.
